# The Statistical Report of King Edward's Hospital Fund—III

**Published:** 1914-01-17

**Authors:** 


### HOSPITAL ECONOMICS.

(Inquiries and Correspondence are invited.)

### The Statistical Report of King Edward's Hospital Fund.*—III.

The examples quoted in the last article point to two tentative conclusions. One is that a hospital is generally more expensive to maintain when it has a medical school attached; the other that outlying hospitals often show a smaller cost per occupied bed than those nearer the centre of London. We will now examine these propositions in the light of more extended data.

Group I. in the Statistical Report is of the larger general hospitals, the definition of this term being those with an average of over 100 occupied beds, for the year 1912. The German Hospital was closed for alterations during the greater part of the twelve months, and no particulars of it are given.

### A Practical Comparison.

Placed in order, beginning with the lowest cost per occupied bed in the first column and the lowest cost per out-patient attendance in the second, the institutions rank as follows:—

| | Cost per Occupied Bed for Twelve Months. | Cost per Out-patient Attendance. |
|---|---|---|
| 1 | Seamen's | West Ham |
| 2 | Prince of Wales's | West London |
| 3 | * University College | * Guy's |
| 4 | West London | London Homœopathic |
| 5 | London Homœopathic | Prince of Wales's |
| 6 | West Ham | Metropolitan |
| 7 | * Middlesex | Seamen's |
| 8 | Great Northern Central | * Middlesex |
| 9 | * Guy's | Gt. Northern Central |
| 10 | * St. Mary's | * St. George's |
| 11 | * St. Thomas's | * Westminster |
| 12 | * Westminster | * St. Mary's |
| 13 | * The London | * St. Thomas's |
| 14 | * Royal Free | * University College |
| 15 | Metropolitan | * The London |
| 16 | * Charing Cross | * Charing Cross |
| 17 | * King's College | * Royal Free |
| 18 | * St. George's | * King's College |

* Hospitals with medical schools.

The cost per occupied bed averaged over seventeen hospitals (St. George's is excluded) is £81 12s. 10d., and the average cost per out-patient attendance 7.24d. As the work of King's College Hospital has not been carried on under normal conditions for some years, we think it only fair to remove its returns from the general calculation, as, necessarily, they are not favourable to the hospital itself and throw up the average of the other sixteen institutions. We now find the bed average to be £81 1s. 7d. and the out-patient average 7.16d.

To return to our list. It will be seen that the hospitals with medical schools appear, both as regards in- and out-patients, to some disadvantage and that the outlying hospitals take a leading place from the point of view of economy. It is true that there are the exceptions one is prepared to find in all such comparisons and the most remarkable of these are University College and the Metropolitan.

Methods of dividing expenditure between in- and out-patients having been touched upon in these columns it will be interesting to regard the table as it would be if the institutions concerned had all been able to calculate their out-patients on the basis of the average cost of the whole group (modified as described above). If this had been done, the Metropolitan, so low in the first column and so high in the second, would find itself in a more central position on each side. The West London would displace University College in the first column.

We notice that while the hospitals with medical schools are sometimes higher and sometimes lower than the average under other headings, they are, with only one exception, all higher under "Salaries and Wages." It will be remembered that in the case of two examples quoted recently one (with a medical school) spent £668 10s. on medical salaries, while another, doing equal work, spent only £194 (a difference of £3 per occupied bed if the cost referred entirely to in-patients).

However carefully expenditure on the medical school is taken out of the general accounts there must be expenses, some almost indefinable, left behind. A larger number of people use the building; the general cost of lighting, heating, cleaning, repairs, water, and other things must therefore be higher. Yet the hospital does not wish to see its school in financial difficulty and is unwilling to burden it with a charge for anything but direct service.

It has been urged that when there are no dressers extra work falls upon the nursing staff and increases the cost of the latter service, but we cannot find any figures in the Statistical Report to justify this contention. To use once more the illustration of Hospitals A and B, the salaries of the nursing department in the hospital with the medical school are £115 higher than in the one without.

### The Financial Advantage of a Medical School.

Any financial advantage of a medical school is on the other side of the ledger. Every year medical men and women are being sent out into the world who in fair and foul weather will stick up for the hospital of their training; all over the country their work carries them, living advertisements of the institution to which they owe their skill. Who can have failed to notice the frequency with which hospitals with medical schools appear in lists of legacies, and to have made the not unreasonable reflection that a fair proportion of these bequests are the result of the cumulative effect of publicity (in which the school has played a large part), or the direct recommendation of a physician, who is often a friend also, of the testator?

* Previous articles appeared on November 29 and December 13, 1913.

With regard to the effect of geographical position on expenditure there is little or no direct evidence. It seems but a phenomenon revealed by the figures of the Report in respect of the larger general and special hospitals; it may be entirely adventitious. However, two of several considerations may be mentioned. The excellent bargain a hospital makes with its honorary medical staff, that in return for certain valuable advantages free medical attendance on the patients will be given, is not always a clear financial gain. A large and eminent staff often means expense in many directions and it often follows that an outlying hospital does not feel the burden of medical requirements so keenly. The other point refers to the experience of most hospital managers, that builders' and decorators' work of all kinds is much more cheaply carried out in the suburbs than in the central parts of London.

Effect of Size on Cost per Bed.

A word may be said here as to the effect of the size of the hospital on the cost per occupied bed. It seems reasonable to put forward the suggestion that a large hospital can and should be run on more economical lines than a small one. Bought on a large scale goods are cheaper, certain standing expenses are almost as high with a hospital of, say, 75 beds as with one of 150, and the larger the institution the more is experience accepted as part of remuneration by the employees. The cost per occupied bed in the larger hospitals (*without* medical schools), average size 144 beds, is appreciably smaller than that of the general hospitals in Group II. of the Report, average size 47 beds, the respective figures being £70 15s. 2d. and £75 18s. 9d. We do not attempt comparison of out-patient figures as several of the smaller hospitals treat none.

Some of the pages of the Statistical Report dealing with the smaller groups of special hospitals are not so helpful as those we have reviewed. It is impossible, for instance, to form any conclusions from a survey of the figures of the Brompton Hospital side by side with three other institutions for consumption of varying sizes, whose beds totalled together only just exceed in number those of their big sister. The same remark may be made of the National Hospital for the Paralysed and Epileptic. The effect of the comparisons is not instructive to a great extent, but for the sake of completeness it is clear that the groups had to be included. But when the statistics of these hospitals take their place in the pages giving general particulars of the whole of the 109 London hospitals compared they are of greater value.

In looking over these general summaries we have indulged in the speculation as to how a philanthropist with a fixed sum to spend on maintaining one particular hospital for one year would best lay out his money.

We hope in our next article to suggest a few improvements to the Statistical Report, and to deal with some subjects not yet touched upon.

